# Ouabain-regulated phosphoproteome reveals molecular mechanisms for Na^+^, K^+^–ATPase control of cell adhesion, proliferation, and survival

**DOI:** 10.1096/fj.201900445R

**Published:** 2019-07-10

**Authors:** Elena Panizza, Liang Zhang, Jacopo Maria Fontana, Kozo Hamada, Daniel Svensson, Evgeny E. Akkuratov, Lena Scott, Katsuhiko Mikoshiba, Hjalmar Brismar, Janne Lehtiö, Anita Aperia

**Affiliations:** * Department of Oncology-Pathology, Science for Life Laboratory, Karolinska Institutet, Solna, Sweden;; † Department of Women’s and Children’s Health, Karolinska Institutet, Solna, Sweden;; ‡ Department of Applied Physics, Science for Life Laboratory, Royal Institute of Technology, Stockholm, Sweden;; § Laboratory for Developmental Neurobiology, Brain Science Institute, Riken, Saitama, Japan

**Keywords:** calcium and calmodulin–dependent protein kinase, phosphoproteomics, apoptosis, inositol triphosphate receptor, kidney

## Abstract

The ion pump Na^+^, K^+^–ATPase (NKA) is a receptor for the cardiotonic steroid ouabain. Subsaturating concentration of ouabain triggers intracellular calcium oscillations, stimulates cell proliferation and adhesion, and protects from apoptosis. However, it is controversial whether ouabain-bound NKA is considered a signal transducer. To address this question, we performed a global analysis of protein phosphorylation in COS-7 cells, identifying 2580 regulated phosphorylation events on 1242 proteins upon 10- and 20-min treatment with ouabain. Regulated phosphorylated proteins include the inositol triphosphate receptor and stromal interaction molecule, which are essential for initiating calcium oscillations. Hierarchical clustering revealed that ouabain triggers a structured phosphorylation response that occurs in a well-defined, time-dependent manner and affects specific cellular processes, including cell proliferation and cell-cell junctions. We additionally identify regulation of the phosphorylation of several calcium and calmodulin–dependent protein kinases (CAMKs), including 2 sites of CAMK type II-γ (CAMK2G), a protein known to regulate apoptosis. To verify the significance of this result, CAMK2G was knocked down in primary kidney cells. CAMK2G knockdown impaired ouabain-dependent protection from apoptosis upon treatment with high glucose or serum deprivation. In conclusion, we establish NKA as the coordinator of a broad, tightly regulated phosphorylation response in cells and define CAMK2G as a downstream effector of NKA.—Panizza, E., Zhang, L., Fontana, J. M., Hamada, K., Svensson, D., Akkuratov, E. E., Scott, L., Mikoshiba, K., Brismar, H., Lehtiö, J., Aperia, A. Ouabain-regulated phosphoproteome reveals molecular mechanisms for Na^+^, K^+^–ATPase control of cell adhesion, proliferation, and survival.

The ubiquitous integral plasma membrane protein Na^+^, K^+^–ATPase (NKA) is well known for its capacity to maintain a steep electrochemical gradient across the plasma membrane by transporting 3 Na^+^ ions out of and 2 K^+^ ions into cells at the expense of 1 molecule of ATP. The minimal functional NKA unit is a heterodimer, consisting of a catalytic α subunit and a supportive β subunit (1). The α subunit is a highly specific receptor for cardiotonic steroids, which consist of a steroid attached to a sugar and an R group that determines their specificity. Cardiotonic steroids include the cardenolide ouabain, which has been identified in mammals (2–4). Ouabain is commonly used to study the NKA function. High concentration of ouabain inhibits the NKA pumping function and will eventually cause cell death, whereas subsaturating concentration stimulates cell proliferation (5–7), modulates cell-to-cell contacts (8, 9), and protects from apoptosis (10–13). Subsaturating concentration of ouabain triggers low-frequency oscillations of intracellular calcium concentration ([Ca^2+^]_i_) and phosphorylation of several signaling molecules, including mitogen-activated protein kinases, protein kinase B, and SRC, in primary and immortalized mammalian cells (14, 15).

Our group has shown that the [Ca^2+^]_i_ oscillatory response to ouabain is initiated *via* direct interaction between the N terminus of the NKA-α subunit, a segment of the molecule that is not essential for ion transport function, and the N terminus of the inositol 1,4,5 triphosphate receptor (16–18). Most studies regarding the [Ca^2+^]_i_ oscillatory response to ouabain have been performed on primary rat kidney epithelial cells, but ouabain-triggered [Ca^2+^]_i_ oscillations are also observed in immortalized COS-7 cells derived from monkey kidney (16, 19). Studies on primary cells have shown that subsaturating concentration of ouabain protects from apoptosis caused by the Shiga toxin, adverse developmental programming, and proteinuric kidney disease. This antiapoptotic effect involves deactivation of proapoptotic B-cell lymphoma 2 (Bcl-2)–family proteins (11–13).

Taken together, our results and others imply that NKA represents a novel class of signaling transducer. It is however still an open question whether ouabain-bound NKA can activate a cascade of intracellular signaling and whether such a signaling cascade affects the regulation of cell adhesion, proliferation, and the apoptotic pathway. To address these questions, we performed a phosphoproteomic study in COS-7 cells treated with a subsaturating concentration of ouabain for 10 and 20 min. We identified 2580 phosphorylation sites (phospho-sites) regulated by ouabain-bound NKA. The phosphorylation of numerous proteins associated with cell adhesion, cell proliferation, and calcium and calmodulin–dependent protein kinases (CAMKs) was prominently altered in response to ouabain treatment.

The family of CAMKs type II (CAMK2s) is specifically sensitive to [Ca^2+^]_i_ oscillations (20, 21). We found the γ subunit of CAMK2 (CAMK2G) to be strongly phosphorylated after ouabain treatment. CAMK2G is reported to have an antiapoptotic effect by deactivating the proapoptotic protein BCL2 associated agonist of cell death (BAD), a member of the Bcl-2 family (22). Subsaturating concentration of ouabain protects primary kidney epithelial cells from apoptosis caused by a variety of stimuli that activate proapoptotic Bcl-2–family proteins (10, 12, 13). To verify the functional significance of CAMK2G phosphorylation, we examined whether CAMK2G down-regulation in primary renal epithelial cells might prohibit the ouabain antiapoptotic effect.

## MATERIALS AND METHODS

### Cell culture and treatment

Monkey (*Cercopithecus aethiops*) kidney cells COS-7 [CRL-1651; American Type Culture Collection (ATCC), Manassas, VA, USA] were cultured in DMEM (D5671; MilliporeSigma, Burlington, MA, USA) supplemented with 10% (v/v) fetal bovine serum (FBS) and 2 mM l-glutamine (all from MilliporeSigma). For all experiments, culture medium was substituted with a new one containing a lower concentration of FBS (0.5% v/v for proteomics and 0.2% v/v for Western blotting) 12 h before treatment with 100 nM ouabain (MilliporeSigma) for 5, 10, or 20 min. For proteomics analysis, untreated and treated cells were exposed to analogous atmospheric and thermal conditions during the time of treatment addition.

Primary culture of rat proximal tubular cells was prepared as previously described in Li *et al.* (10). Cells were seeded in 24-well plates containing 12-mm coverslips or in 6-well plates and maintained in low-glucose DMEM (31600083; Thermo Fisher Scientific, Waltham, MA, USA) supplemented with 10% (v/v) FBS, 4 mM NaHCO_3_, 10 µg/ml penicillin, and 10 µg/ml streptomycin. Experiments were performed at day 3 of culture, when 99% of the cells were rat proximal tubular cells.

### Calcium recording

For calcium recordings, COS-7 cells were seeded on 18-mm coverslips and transfected with GCaMP6, a genetically encoded calcium indicator, the day before the experiment. The coverslips with cells were mounted in a perfusion chamber, and time-lapse recordings were made using a Zeiss LSM 510 confocal microscope (Carl Zeiss, Oberkochen, Germany) with an excitation wavelength of 480- and 510-nm long-pass detection. Spectral analysis of recorded [Ca^2+^]_i_ oscillations was performed with Matlab software (MathWorks, Natick, MA, USA) as previously described in Zhang *et al.* (18). The Ca^2+^ release-activated Ca^2+^ channel inhibitor BTP-2 (203890; Calbiochem, San Diego, CA, USA) was used at a 50-μM concentration. Krebs-Ringer bicarbonate buffer was used for perfusion and washout. Measurements were repeated in 4 independent preparations and 6–8 individual cells were examined within each visual field.

### Sodium imaging

For sodium recording, cells were seeded onto 18-mm coverslips and loaded with 5 μM of the sodium-sensitive dye Asante NaTrium Green-2 acetoxymethyl (AM) (TefLabs, Austin, TX, USA) for 30 min at 37°C. Fluorescence of Asante NaTrium Green-2 AM was excited at 490 nm and collected above 510 nm. Cells were then perfused with physiologic solution [110 mM NaCl, 25 mM NaHCO_3_, 4 mM KCl, 1.2 mM MgCl_2_, 1 mM NaH_2_PO_4_, 1.5 mM CaCl_2_, 10 mM glucose, and 20 mM HEPES (pH = 7.4)] followed by addition of 100 nM ouabain. At the end of the experiment, cells were perfused with physiologic solution containing 3 μM gramidicin (G5002; MilliporeSigma) and 10 μM monensin (M5273; MilliporeSigma) to permeabilize cells and increase intracellular sodium concentration nominally up to 136 mM, as in perfusion solution. Measurements were repeated in 4 independent preparations and 2–4 individual cells were examined within each visual field.

### Protein extraction for proteomics

For protein extraction, culture dishes were rinsed twice with ice-cold PBS and cells were lysed by scraping in the presence of 0.5% (w/v) sodium deoxycholate, 0.35% (w/v) sodium lauroyl sarcosinate, 1 mM DTT, and Halt Protease and Phosphatase Inhibitor Cocktail (Thermo Fisher Scientific, Waltham, MA, USA). Lysates were heated at 95°C for 10 min prior to sonication followed by centrifugation for 15 min at 14,000 *g* and 4°C. Supernatants were transferred to new vials, and protein levels were quantified using the DC Protein Assay (Bio-Rad, Hercules, CA, USA).

### Protein digestion

The protein extracts were processed following the filter-aided sample preparation protocol (23) with slight modifications. Briefly, protein extracts were applied on 10k filtration units (MilliporeSigma) and reduced with 1 mM DTT in the presence of 8 M urea and 50 mM HEPES. Upon centrifugation, proteins were alkylated with 5.5 mM iodoacetamide in the presence of 4 M urea and 50 mM HEPES and centrifuged again. Subsequently, proteins were washed once before digestion at 37°C for 4 h with Lys-C 1:50 w/w (Wako Pure Chemicals, Tokyo, Japan), both in the presence of 0.5 M urea and 50 mM HEPES. Finally, a 50-mM HEPES solution containing trypsin 1:25 (w/w) (Thermo Fisher Scientific) was added and samples were incubated overnight at 37°C. The peptides were collected by centrifugation of the filter-aided sample preparation filters, and the samples were desalted using polymeric Reversed Phase–Solid Phase Extraction cartridges (Phenomenex, Torrance, CA, USA). Peptide concentration was measured with DC Protein Assay and separate 500 and 50 μg aliquots of peptides/sample were lyophilized and set aside for phosphoproteomics and proteomics analysis, respectively.

### Phosphorylated peptide enrichment

Phosphorylated peptide (phosphopeptide) enrichment was performed using Stop And Go Extraction tips loaded with titanium dioxide beads (GL Sciences, Tokyo, Japan) as previously reported (24, 25). After binding the peptides to the beads and washing 3 times, phosphopeptides were eluted with 2 × 100 μl of 100 μl 3% (v/v) triethylamine buffer into vials containing 200 μl of 10% (v/v) formic acid to neutralize the pH. Triethylamine buffer is advantageous for phosphopeptide elution compared with ammonia buffer because the triethylamine buffer is volatile and does not require sample desalting, thereby avoiding sample losses connected with this cleaning step. Eluates were lyophilized prior to tandem mass tag (TMT) 10-plex labeling (Thermo Fisher Scientific).

### TMT labeling

Phosphopeptide and peptide samples were labeled with 10-plex TMT reagents as previously described in Panizza *et al.* (24). The efficiency of labeling was verified by liquid chromatography tandem mass spectrometry (LC-MS/MS) before pooling samples. Pooled samples were desalted with Reversed Phase–Solid Phase Extraction cartridges and then lyophilized in a SpeedVac (Thermo Fisher Scientific) before focusing on immobilized pH gradient (IPG) gel strips (GE Healthcare, Waukesha, WI, USA).

### Peptide-level high-resolution isoelectric focusing

High-resolution isoelectric focusing (HiRIEF) was performed as previously described in Branca *et al.* (26) using linear pH ranges of 2.5–3.7 (ultra-acidic range, a strip prototype provided by GE Healthcare) (24) or 3–10 (wide-range, commercially available from GE Healthcare). The sample containing TMT-labeled phosphopeptides was split in 2 and prefractionated using the ultra-acidic and wide-range IPG strips (Phospho HiRIEF). The sample not enriched for phosphopeptides was fractionated using the wide-range IPG strip (Standard HiRIEF). Strips were divided into 72 fractions (fraction numbering proceeds from the strip acidic end to the basic end) and extracted to V-bottom 96-well plates with a liquid handling robot (GE Healthcare prototype modified from Gilson liquid handler 215). Plates were lyophilized in a SpeedVac prior to LC-MS/MS analysis.

### LC-MS/MS

For Phospho HiRIEF, all 72 fractions from the ultra-acidic IPG strip plate along with the first 55 fractions from the wide-range IPG strip plate were analyzed with LC-MS/MS. For Standard HiRIEF, all 72 fractions from the wide-range IPG strip plate were analyzed with LC-MS/MS.

Each HiRIEF fraction was dissolved in 15 µl of phase A (95% water, 5% DMSO, and 0.1% formic acid), mixed by drawing and dispensing 10 µl 10 times, followed by the autosampler (Ultimate 3000 Rapid Separation Liquid Chromatography System; Thermo Fisher Scientific) injecting 10 µl into a C18 guard desalting column (Acclaim PepMap 100; 75 µm × 2 cm, nanoViper; Thermo Fisher Scientific). Following 5 min of flow at 5 µl/min driven by the loading pump, the 10-port valve switched to analysis mode, in which the binary high-pressure gradient pump (referred to as nanogradient pump) provided a flow of 250 nl/min through the guard desalting column. From an initial composition of 3% phase B (90% acetonitrile, 5% DMSO, 5% water, and 0.1% formic acid), the reversed-phase gradient proceeded to 45% phase B over 50 min. Upon completion of the gradient, the column was washed with a solution of 99% phase B for 10 min and re-equilibrated to the initial composition. Total LC-MS/MS run time was 74 min. A nano Easy-Spray column (PepMap Rapid Separation Liquid Chromatography; C18; 2-µm bead size; 100Å pore size; 75-µm internal diameter; 50 cm long; Thermo Fisher Scientific) was used on the nanoelectrospray ionization Easy-Spray source at 60°C. Online LC-MS/MS was performed using a hybrid Q Exactive mass spectrometer (Thermo Fisher Scientific). Fourier transform–based mass spectrometer (FTMS) master scans with a resolution of 70,000 (and mass range 300–1700 *m*/*z*) were followed by data-dependent MS/MS (35,000 resolution) on the 5 most abundant ions using higher-energy collision dissociation (HCD) at 30% normalized collision energy. Precursor ions were isolated with a 2 *m*/*z* window. Automatic gain control targets were 1 × 10^6^ for MS1 and 1 × 10^5^ for MS2. Maximum injection times were 100 ms for MS1 and 150 ms (for proteomics) or 400 ms (for phosphoproteomics) for MS2. The entire duty cycle lasted ∼1.5 s. Automated precursor-ion dynamic exclusion was used with a 60 s duration. Precursor ions with unassigned charge states or a charge state of +1 were excluded. An underfill ratio of 1% was applied.

### Proteomics database search

All MS/MS spectra were searched by Sequest/Percolator under the Proteome Discoverer software platform (PD v.1.4; Thermo Fisher Scientific) using a target-decoy strategy. The reference database was the *Chlorocebus sabaeus* protein subset of Uniprot (release 2017-04-05, 16,415 entries). Precursor-ion and product-ion mass tolerances of 10 ppm and 0.02 Da, respectively, were used for HCD-FTMS. Additionally, peptide spectral matches allowed for up to 2 missed trypsin cleavages (Lys-Pro and Arg-Pro were not considered cleavage sites). Carbamidomethylation on cysteine and TMT 10-plex on lysine and the N terminus were set as fixed modifications, and oxidation of methionine was set as a dynamic modification while searching all MS/MS spectra. Phosphorylation of serine, threonine, and tyrosine were included as dynamic modifications while searching MS/MS spectra from the Phospho HiRIEF LC-MS/MS analysis. Quantitation of TMT 10-plex reporter ions was performed using an integration window tolerance of 10 ppm. A false discovery rate cutoff of 1% was applied at the peptide level. The phosphoRS algorithm node was added to the workflow for the phosphoproteomics search to obtain probabilities of localization of phospho-sites (27), and only sites with a high confidence of localization (≥95 pRS score) were used for quantification.

### Ratio calculation and definition of significantly regulated events for phosphoproteomics and proteomics data

For both Phospho HiRIEF LC-MS/MS and Standard HiRIEF LC-MS/MS, only peptide spectral matches where the intensity of the TMT reporter ions was measured for all the 10 samples were employed for ratio calculation. Ratios were calculated as previously described in Panizza *et al.* (24), with minor modifications. Both protein and phospho-site ratios were normalized to the median of all ratios / TMT channel, assuming equal peptide and phosphopeptide loading of all 10 samples. Because the protein levels displayed only minor changes at the time points employed for treatment, phospho-site ratios were not normalized to changes in protein abundance. Thresholds to define significant regulation upon ouabain treatment were set based on the distribution of the phospho-site and protein ratios in the untreated samples. Thresholds for down-regulation were set as the average of the fifth percentile of the 4 untreated sample ratio distributions, and thresholds for up-regulation were the average of the 95th percentile of the 4 untreated sample ratio distributions (Supplemental Fig. S1). Events regulated with a fold-change above or below the thresholds and with a *P*-value lower than 0.01 were deemed significant.

### Western blot analysis

Cells were lysed in RIPA buffer [125 mM Tris, 154 mM NaCl, 1% NP-40, 6 mM sodium deoxycholate, and 1.3 mM EDTA (pH = 7.4)] supplemented with Pierce protease and phosphatase inhibitor mini tablet (88669; Thermo Fisher Scientific). Protein concentration was measured using the DC Protein Assay, and loading buffer was added to the sample to a final protein concentration of 1 µg/µl. Samples were heated at 95°C for 5 min prior to loading on the electrophoresis gel. Electrophoresis and transfer were performed employing Bio-Rad systems. Membranes were blocked for 1 h in 5% (w/v) bovine serum albumin dissolved in 1× Tris-buffered saline solution. Primary and secondary antibodies were diluted in 1× Tris-buffered saline solution containing 0.2% (v/v) Tween-20 and 5% bovine serum albumin (w/v). The following antibodies were employed: phosphorylated ERK (mouse; 1:200; sc-7383; Santa Cruz Biotechnology, Dallas, TX, USA), α-tubulin (mouse; 1:2000; T6074; MilliporeSigma), CAMK2G (rabbit; 1:500; ab37999; Abcam, Cambridge, MA, USA), glyceraldehyde 3-phosphate dehydrogenase (mouse; 1:1000; sc-32233; Santa Cruz Biotechnology), horseradish peroxidase–conjugated anti-mouse IgG (sheep; 1:4000; NXA931; GE Healthcare), and horseradish peroxidase–conjugated anti-rabbit IgG (donkey; 1:4000; NA934V; GE Healthcare). Images were acquired and analyzed using an Odyssey Imaging system from Li-Cor Biosciences (Lincoln, NE, USA).

### Cluster extraction and gene ontology enrichment analysis

For all analyses, plots were generated using RStudio (Boston, MA, USA). Clusters representing significantly regulated phospho-sites were extracted from up-regulated and down-regulated phospho-sites separately (3 clusters from each) by cutting the row dendrograms based on height. Gene ontology (GO) enrichment analysis was performed with the web service GOrilla (*http://cbl-gorilla.cs.technion.ac.il*) by selecting the “two unranked lists of genes” option with the list of all genes identified in the proteomic analysis as background set. The *P*-value threshold was set at 10^−3^. The top enriched GO terms, and with a fold enrichment of at least 2 folds and *P* value <0.01, are displayed. A list of phospho-sites with regulatory functions was obtained using the “Regulatory_sites” file available from the PhosphoSitePlus database (*https://www.phosphosite.org*) (28).

### Protein class and phosphorylation motif enrichment analyses

Genes were assigned to different classes using publicly available databases for protein kinases (29), protein phosphatases (30), transcription factors (31, 32), and enzymes belonging to the ubiquitin and ubiquitin-like conjugation systems (33). GO annotations containing “calcium” or “calmodulin” within the term definition were employed for selecting calcium and calmodulin–dependent proteins. A complete list of the GO terms employed in this analysis is reported in Supplemental Data Set S4.

Enriched phosphorylation motifs (phospho-motifs) were extracted using motif-x (*http://motif-x.med.harvard.edu*) (34). Sequence windows for phospho-sites that are significantly regulated in each cluster and for all the identified phospho-sites were used as foreground and as background data sets, respectively. The “occurrence” parameter was set at 20 for serine phospho-sites and at 10 for threonine phospho-sites; the “significance” parameter was set at 0.00001, “width” at 15, and “foreground format” and “background format” as “prealigned” (±7 residues around the identified phospho-site). Enrichment analysis was not performed for tyrosine phospho-sites because of the low number of significantly regulated tyrosine phospho-sites. Putative kinases for each motif were annotated using information available from the Human Protein Reference Database (HPRD; *http://www.hprd.org*) (35) as well as from other published resources (36, 37).

### Protein-protein interaction network analysis

Protein-protein interaction networks were generated using the online tool provided by the Search Tool for the Retrieval of Interacting Genes/Proteins (STRING; *https://string-db.org*) (38). Only the highest confidence interactions (interaction score >0.900) were considered for the analysis.

### Modeling the structure of inositol triphosphate receptor type 3 around S1832

The position of S1832 within inositol triphosphate (InsP_3_) receptor type 1 revealed its localization within the α-helical domain 3, which is essential to mediate the opening of the calcium channel of the InsP_3_ receptor (39) (Supplemental Fig. S3*A*). The amino acid sequences of several isoforms of the InsP_3_ receptor were aligned using the Clustal X (*http://www.clustal.org/clustal2/*) program to localize the position of the S1832 residue of the type 3 receptor with respect to the receptor type 1 protein domains (Supplemental Fig. S3*B*). The X-ray model of InsP_3_ receptor type 1 [Protein Data Bank identifier (PDB ID) 5X9Z (39)] was employed to analyze the position of the S1832 residue within the tridimensional structure of the protein.

### Transfection

Lipofectamine RNAImax Transfection Reagent (13778075; Thermo Fisher Scientific) and Opti-MEM I Reduced Serum Medium (31985062; Thermo Fisher Scientific) were used for small interfering RNA (siRNA) transfection according to the manufacturer’s instructions. Stealth siRNA (RSS332172; Thermo Fisher Scientific) was employed for CAMK2G silencing, and Stealth RNAi siRNA Negative Control, Med GC (12935300; Thermo Fisher Scientific) was used for controls. Cells were transfected with 20 nM of construct for 48 h before treatments.

### Apoptosis measurement

After transfection with CAMK2G siRNA or negative control as described above, primary rat proximal tubular cells were cultured with 10% (v/v) or 0.2% (v/v) FBS in the presence or absence of 10 nM of ouabain for 24 h or cultured in normal (5.5 mM) or high (20 mM) glucose concentration, in the presence or absence of 10 nM ouabain for 6 h. Rat proximal tubular cells were fixed in ice-cold methanol for 5 min before permeabilization with 33% (v/v) acetic acid in ethanol for 5 min at −20°C. Cells were then repeatedly washed with PBS. Apoptosis was detected using an ApopTag Red *In Situ* Apoptosis Detection Kit (S7165; MilliporeSigma), according to the manufacturer’s instructions. ApopTag is an indirect terminal deoxynucleotidyl transferase dUTP nick end labeling (TUNEL) method. After staining, coverslips were mounted and imaged in a Zeiss LSM 510 confocal microscope equipped with a ×25 and 0.8 numerical aperture objective. DAPI was detected by excitation at 405 nm and detection at 420–480 nm. TUNEL labeling was detected by excitation at 543 and 575 nm long-pass detection. For each of the coverslips, 6 images were recorded and an average apoptotic index (percentage of apoptotic cells from total number of cells) was calculated.

### Statistics for Western blot and TUNEL assay quantification

Summarized data are presented as means ± sem. Each culture well represents 1 biologic replicate (*n* = 1). Statistical significance for multiple comparisons was calculated using ANOVA followed by Tukey’s test for *post hoc* analysis, unless otherwise indicated.

### Data availability

The MS proteomics data have been deposited into the ProteomeXchange Consortium *via* the Proteomics Identifications (PRIDE) partner repository (*http://www.ebi.ac.uk/pride*) with the data set identifier PXD010006. All other data are available in the manuscript and as Supplemental Information, or from the corresponding author upon reasonable request.

### Ethical statement

All the work involving animals was performed in compliance with Karolinska Institutet regulations concerning care and use of laboratory animals. All experiments were approved by the Stockholm North Ethical Evaluation Board for Animal Research (ethical permit no. N212/14).

## RESULTS

### Ouabain treatment leads to extensive changes in the phosphoproteome of COS-7 cells

**Figure 1:**
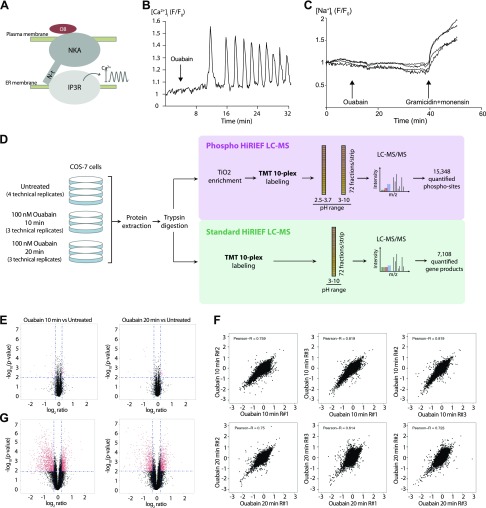
Treatment with ouabain for 10 and 20 min mediates extensive changes of the phosphoproteome of COS-7 cells. *A*) Schematic representation of the interaction between NKA and InsP_3_ receptor (IP3R) upon ouabain binding to NKA. The interaction leads to the release of calcium ions from the ER. *B*) Representative measurement of the intensity of the fluorescence emitted by the calcium-sensitive dye GCaMP6 in a single cell over time. F/F_0_, fluorescence signal:fluorescence at *t* = 0; OB, ouabain. The arrow indicates the time point of ouabain addition. *C*) Representative measurement of the intensity of the fluorescence emitted by the sodium-sensitive dye Asante NaTrium Green-2 AM in single cells over time. Arrows indicate the time points of addition of the indicated drugs. *D*) Experimental workflow employed for phosphoproteomic and proteomic analyses [Phospho HiRIEF LC-MS and Standard HiRIEF LC-MS (24), respectively]. The number of phospho-sites and proteins identified and quantified is displayed. TiO_2_, titanium dioxide. *E*) Volcano plots representing significantly regulated events in the proteomic analysis (red dots). Left and right panels display the 10-min and the 20-min time points, respectively. The average fold-change of the 3 replicates for each experimental condition is represented on the *x* axis, and the significance of the regulation based on Student’s *t* test is represented on the *y* axis. *F*) Correlation of phospho-site log_2_-transformed ratio values for each replicate pair; Pearson correlation coefficient is displayed. Ratio values are calculated using the average of the 4 untreated samples as a denominator. R#, replicate no. *G*) Volcano plots representing significantly regulated events in the phosphoproteomic analysis (red dots). The average fold-change of the 3 replicates for each experimental condition is represented on the *x* axis, and the significance of the regulation based on Student’s *t* test is represented on the *y* axis.

Our previous work demonstrated that treatment of primary rat proximal tubular cells with subsaturating concentration of ouabain activates the InsP_3_ receptor and triggers [Ca^2+^]_i_ oscillations and that the release of calcium from the endoplasmic reticulum (ER) in response to ouabain depends on the physical association between the InsP_3_ receptor and NKA (**Fig. 1*A***) (16–18). Here, we employed COS-7 cells, which display an oscillatory [Ca^2+^]_i_ response to ouabain similar to that of primary proximal tubular cells, to uncover the signaling network activated by ouabain-bound NKA. Regular [Ca^2+^]_i_ oscillations were observed ∼10 min after the application of 100 nM ouabain and were sustained for at least 20 min (Fig. 1*B*). The intracellular sodium concentration remained constant during this time (Fig. 1*C*), indicating that 100 nM ouabain does not impair the capacity of NKA to maintain the gradient of Na^+^ ions across the plasma membrane through the treatment period. The ionophores gramicidin and monoensin were added as a negative control, to ablate the electrochemical gradient maintained by the NKA by allowing a rapid inflow of Na^+^ ions into the cells.

To characterize the early downstream effects of the [Ca^2+^]_i_ oscillations mediated by the ouabain-NKA-InsP_3_ receptor, phosphoproteomic and proteomic analyses of COS-7 cells were performed on untreated cells (four replicates) and cells treated with 100 nM ouabain for 10 and 20 min (three replicates each). Phosphoproteomic analysis was performed according to a workflow that we recently developed that provides for accurate multiplex quantification, high analytical depth, and requires a low amount of starting material (Fig. 1*D*) (24). The approach led to the identification and quantification of 15,348 phospho-sites, corresponding to 3937 protein-coding genes (Supplemental Data Set S1 and Supplemental Table S1). Proteomic analysis identified 7108 protein-coding genes (Supplemental Data Set S2 and Supplemental Table S1). Phosphoproteomic and proteomic analyses are based on gene-centric quantification, thus protein-coding genes are denoted proteins henceforth. For both phosphoproteomics and proteomics analyses, the quantification for each sample (untreated and treated) is expressed as a ratio relative to the average of the 4 untreated samples. Phospho-sites and proteins that have ratios above certain thresholds (defined in Supplemental Fig. S1) and are consistent across replicates are considered to be significantly regulated by ouabain treatment.

Proteomic analysis reveals that minor changes of total protein levels occur upon 10 and 20 min ouabain treatment, as shown by the tight distribution of protein ratios around zero (Supplemental Fig. S2). In particular, only 80 and 48 proteins are significantly regulated after 10 and 20 min of treatment, respectively (Fig. 1*E* and Supplemental Data Set S2, Sheet 2). In contrast, the phospho-site ratios have a wide distribution, indicating that a large number of phosphorylation and dephosphorylation events occur in response to ouabain treatment and show good reproducibility (Pearson correlation coefficients for every pair of replicates range between 0.61 and 0.82, Fig. 1*F*). Specifically, 1941 phospho-sites (86.1% phosphorylated Ser, 13.7% phosphorylated Thr, and 0.3% phosphorylated Tyr) and 1484 phospho-sites (82.5% phosphorylated Ser, 17.0% phosphorylated Thr, and 0.5% phosphorylated Tyr) are significantly regulated after 10 and 20 min of treatment, respectively (Fig. 1*G* and Supplemental Data Set S1, Sheet 2).

### Ouabain regulates proteins responsible for the oscillatory calcium signal

**Figure 2:**
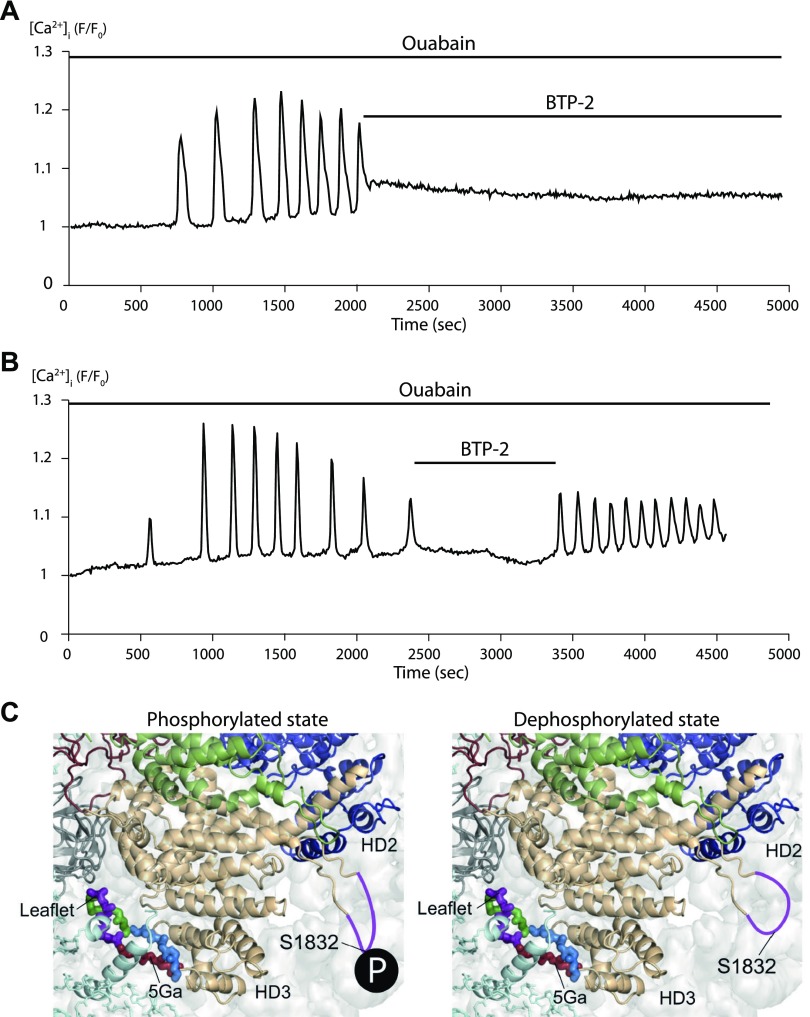
STIM and the InsP_3_ receptor play central roles in mediating ouabain-dependent [Ca^2+^]_i_ oscillations. *A*, *B*) Recordings of the intensity of the fluorescence emitted by the calcium-sensitive dye GCaMP6 in single cells over time. Time periods when drugs were applied are indicated by solid horizontal lines. The measurements are representative of those obtained from 6 to 8 individual cells identified within each field of view and repeated in 4 different preparations performed on different days. F/F_0_, fluorescence signal:fluorescence at *t* = 0. *C*) Simulation of the structural rearrangement induced by dephosphorylation of S1832 of InsP_3_ type 1 receptor (right panel) compared with the phosphorylated form of the protein (left panel), based on the crystal model with PDB ID 5X9Z (39). Purple coloring represents the receptor loop region. 5Ga, region of 5 amino acids mutated in the orginal study (39, red-colored); HD2, α-helical domain 2; HD3, α-helical domain 3.

An oscillatory calcium signal requires activation of both the stromal interaction molecule (STIM) and the InsP_3_ receptor. STIM senses a decrease in calcium concentration in the ER and activates plasma membrane calcium channels to replenish the ER calcium stores following calcium outflow *via* the InsP_3_ receptor (40). Treatment with BTP-2, a potent inhibitor of STIM1-coupled Ca^2+^ release-activated Ca^2+^ channel–mediated Ca^2+^ entry, abolishes ouabain-triggered [Ca^2+^]_i_ oscillations (**Fig. 2*A***), and oscillations are restored upon removal of the drug (Fig. 2*B*). Phosphoproteomic analysis identified 3 up-regulated phospho-sites of STIM1, including S575, which is known to be involved in STIM activation following the depletion of ER calcium stores (41, 42). Additionally, 1 phospho-site on the ubiquitously expressed InsP_3_ receptor type 3 (S1832) is significantly down-regulated (1.3-fold down) after 20 min of ouabain treatment. Using the InsP_3_ type 1 receptor crystal structure [PDB ID 5X9Z (39)], we predict that dephosphorylation at this S1832 site determines a structural rearrangement that disorders the loop region, increasing the opening probability of the calcium channel of the InsP_3_ receptor (Fig. 2*C* and Supplemental Fig. S3).

### Temporal analysis of phosphorylation events reveals the role of ouabain signaling for regulation of cell junctions and proliferation

**Figure 3:**
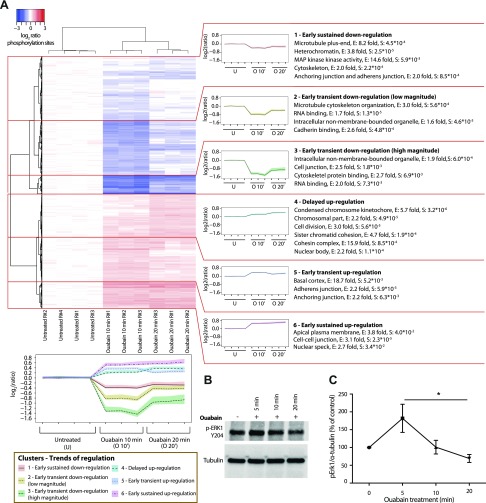
NKA triggers temporally regulated phosphorylation events impacting cell adhesion and proliferation. *A*) Heatmap representing Ward’s method hierarchical clustering based on Euclidian distance of the significantly regulated phospho-site ratios. Line plots (bottom left panel) display the average phospho-site log_2_-transformed ratio across experimental conditions for each of the identified clusters. Color shading represents the 25th and 75th percentile of the distribution of all the phospho-site ratios in the cluster. For each enriched GO term (right panel), E indicates the fold enrichment, and S indicates the *q*-value representing the significance of the enrichment. O, ouabain; U, untreated; R#, replicate no. *B*) Representative images of Western blot analyses of phosphorylated ERK1 (p-ERK1) Y204 and tubulin levels in COS-7 cells protein extracts. *C*) Line plot representing the average level of p-ERK1 Y204 normalized to tubulin in ouabain-treated *vs.* untreated cells. Error bars represent se of the 3 replicates’ means. *P < 0.05.

Hierarchical clustering was applied to the significantly regulated phosphorylation events (2580 phospho-sites), identifying 6 main clusters with characteristic temporal regulation patterns (early or late, and transient or sustained phosphorylation; **Fig. 3*A***, left panel). GO enrichment analysis was employed to examine whether phosphorylation events characterized by different temporal patterns regulate distinct cellular processes (Fig. 3*A*, right panel). Clusters 1, 3, 5, and 6 are enriched in proteins regulating anchoring and adherens cell junctions, which display a high degree of connectivity in a protein interaction network (Supplemental Fig. S4*A*). Previous studies by Cereijido *et al*. demonstrated that ouabain, when employed at a subsaturating concentration, plays a major role in regulating the permeability and composition of tight junctions (8, 9, 43). Furthermore, cell adhesion molecules play a significant role in cancer progression and metastasis, and many of the adhesive molecules phosphorylated in response to ouabain have been suggested as potential therapeutic targets in cancer (44, 45). Our findings will offer further insights into the molecular mechanisms whereby ouabain regulates cell adhesion.

Numerous studies performed in a variety of cell types have shown that ouabain stimulates cell proliferation (5–7, 46, 47). Cluster 4, containing delayed up-regulated phospho-sites, is characterized by the enrichment of proteins involved in cell division. The DNA-dependent protein kinase (PRKDC) is one of the top up-regulated phosphorylated proteins (phosphoproteins) in cluster 5 (Supplemental Data Set S1, Sheet 2). Three of the regulated phospho-sites of PRKDC [T2615, T2641, and T2650 (corresponding to Y2612, T2638, and T2647 in humans)] have been reported to regulate cellular growth (48, 49). PRKDC also plays a role for DNA repair in cells undergoing apoptosis (50). Protein interaction analysis of ouabain-regulated phosphoproteins that are involved with cell proliferation highlights a tight cluster of proteins belonging to the cohesin complex (Supplemental Fig. S4*B*). This complex holds sister chromatids together during mitosis and regulates chromosome segregation, suggesting that ouabain impacts cell cycle progression by regulating progression from mitosis.

### Temporal down-regulation of MAPKs

Analysis of the phospho-sites annotated with the GO term “MAP kinase kinase activity” (enriched in cluster 1, Fig. 3*A*, right panel), reveals that multiple phospho-sites of MAPKs are dephosphorylated 10 and 20 min after ouabain treatment (T185/Y187 of MAPK1, T202/Y204 of MAPK3, and Y182 of MAPK14; **Table 1**). This was an unexpected finding, because numerous reports demonstrate the ability of ouabain to trigger phosphorylation of MAPKs (6, 51–53). However, it should be noted that the current study provides snapshots of ongoing phosphorylation and dephosphorylation processes and that reversible phosphorylation events that occur during the first minutes following ouabain administration were not recorded. To test whether, in fact, phosphorylation of MAPKs occurs at earlier time points than those employed for the phosphoproteomic analysis, we examined MAPK3 (ERK1) phosphorylated Y204 levels over time by Western blot. We indeed found increased Y204 phosphorylation 5 min after ouabain treatment and confirmed its dephosphorylation after 20 min of ouabain treatment (Fig. 3*B, C*). Furthermore, although phosphorylation of MAPKs in response to ouabain is often linked to phosphorylation of SRC (14, 15), no significant regulation of SRC phosphorylation was recorded under the experimental conditions employed for this analysis. We did, however, identify phosphorylation of epidermal growth factor receptor S1026 (Table 1), which mediates inhibition of kinase enzymatic activity as one of the top up-regulated events 10 min after ouabain treatment (1.7-fold up-regulation).

**TABLE 1:** Protein kinases’ functional phospho-sites regulated by ouabain

Gene name	Phospho-site sequence window (±7 aa)	Phospho-site in monkey	Ouabain 10 min - average log_2_(ratio)	Ouabain 20 min - average log_2_(ratio)	Effect on kinase enzymatic activity	Cluster
*MAP2K1*	LIDSMANSFVGTRSY	S222	−0.31	−0.35	inactivation	1
*MAPK1*	HTGFLTEYVATRWYR	Y187	−0.24	−0.65	inactivation	1
*MAPK1*	HDHTGFLTEYVATRW	T185	−0.17	−0.68	inactivation	1
*MAPK1*	HTGFLTEYVATRWYR	Y187	−0.17	−0.68	inactivation	1
*MAPK14*	TDDEMTGYVATRWYR	Y182	−0.28	−0.51	inactivation	1
*MAPK3*	HTGFLTEYVATRWYR	Y204	−0.22	−0.52	inactivation	1
*MAPK3*	HDHTGFLTEYVATRW	T202	0.02	−0.47	inactivation	1
*MAPK3*	HTGFLTEYVATRWYR	Y204	0.02	−0.47	inactivation	1
*PRKACA*	EEEEIRVSINEKCGK	S342	−0.54	−0.27	inactivation	1
*RPS6KB1*	GSPRTPVSPVKFSPG	S447	−0.48	−0.17	inactivation	1
*RPS6KB1*	RFIGSPRTPVSPVKF	T444	−0.48	−0.17	inactivation	1
*CAMKK1*	KSMLRKRSFGNPFEP	S458	−0.70	−0.54	activation	2
*CDK16*	SRRLRRVSLSEIGFG	S227	−0.76	−0.76	activation	2
*GSK3B*	RGEPNVSYICSRYYR	Y216	−0.55	−0.39	inactivation	2
*EGFR*	PQQGFFSSPSTSRTP	S1026	0.76	0.43	inactivation	5
*MAPK7*	DPLPPVFSGTPKGSG	S729	0.23	0.29	inactivation	5
*MAPK7*	LPPVFSGTPKGSGAG	T731	0.23	0.29	inactivation	5

Protein kinases whose enzymatic activity is regulated by ouabain treatment are listed. “Effect on kinase enzymatic activity” column indicates the ultimate effect of the observed regulation, depending on whether the corresponding phospho-site induces or inhibits protein kinase enzymatic activity. A detailed list of references can be found for each of the listed phospho-sites on the PhosphoSitePlus website (28). “Cluster” column corresponds to those defined in Fig. 3. CDK16, cyclin dependent kinase 16; EGFR, epidermal growth factor receptor; GSK3B, glycogen synthase kinase-3B; MAP2K1, mitogen-activated protein kinase kinase 1; PRKACA, protein kinase CAMP-activated catalytic subunit α; RPS6KB1, ribosomal protein S6 kinase B1.

### Ouabain regulates multiple protein kinases

**Figure 4:**
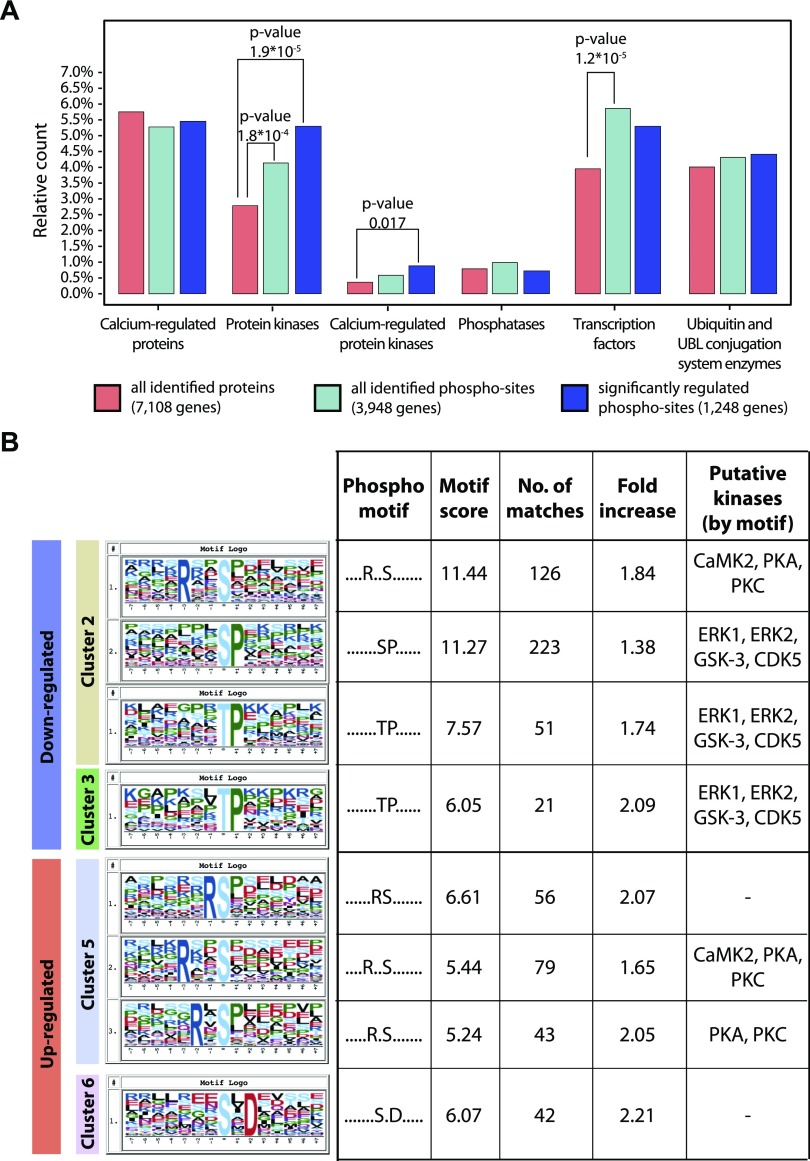
Ouabain modulates the activation status of several protein kinases. *A*) Relative number of genes identified for each of the indicated classes. Protein class enrichment is evaluated by Fisher’s exact test. UBL, ubiquitin-like. *B*) Enrichment of phospho-motifs for each of the identified cluster of regulation, evaluated using motif-x. Motif score represents the significance of the enrichment (negative log_2_-transformed *P* value). No. of matches indicates the number of phospho-sites containing the motif in the subset of significantly regulated phospho-sites. Fold increase indicates the fold enrichment of the motif in the subset of significantly regulated phospho-sites, as compared with the background (all identified phospho-sites). Putative kinases are indicated based on known kinase recognition motifs (35–37). CDK5, cyclin dependent kinase 5; GSK-3, glycogen synthase kinase-3.

To further examine the effects on cellular functions of the phosphosignaling triggered by ouabain, we evaluated the modulation of phospho-sites corresponding to several classes of proteins with regulatory or enzymatic activity. An enrichment of CAMKs is found specifically among the phosphoproteins regulated by ouabain-bound NKA, revealing a marked effect of NKA on this class of proteins. No enrichment of protein phosphatases, transcription factors, or ubiquitin and ubiquitin-like conjugating system enzymes is found (**Fig. 4*A***). To elucidate which kinases are central in mediating the phosphosignaling triggered by ouabain-bound NKA, we looked for kinase-specific motifs that are enriched in each of the clusters of regulated phospho-sites (Fig. 4*B*, left panel). Phospho-motifs associated with CAMKs, with the cAMP-dependent PKA, and with PKC are enriched in clusters 2 and 5. Phospho-motifs associated with MAPK3 (ERK1), MAPK1 (ERK2), glycogen synthase kinase-3, and cyclin-dependent kinase 5 are enriched in clusters 2 and 3.

The CAMKs whose phosphorylation is significantly regulated by ouabain-bound NKA are listed in Supplemental Data Set S3. Among those, we identify the protein kinase Abelson protooncogene 2, which displays 6 phospho-sites regulated upon ouabain treatment. The Abelson family of protein kinases are of critical importance for regulating and maintaining tight junctions (54). Additionally, we find increased phosphorylation of WNK1 S1636, S2539, and S167 upon 20 min of ouabain treatment. WNK1 is a calcium-dependent protein kinase of conceivable physiologic relevance because it is essential for the regulation of electrolyte homeostasis and blood pressure (55) and has been implicated in the genetic disease familial hypertension, which is associated with increased sodium renal reabsorption and hypertension (56). Studies of the long-term effects of ouabain treatment on the blood pressure of rodents resulted in divergent results, which may be explained by differences in salt intake (57). We also found ouabain-dependent regulation of the CAMKs CAMKK1 and CAMK2G, which have both been implicated in the regulation of the intrinsic apoptotic pathway (22, 58). CAMKK1 was dephosphorylated at S458, a site that mediates inactivation of its kinase enzymatic activity (59) (Table 1). CAMK2G was phosphorylated at S402 and S449, whose functions are currently not known.

### CAMK2G plays an essential role for ouabain down-regulation of the intrinsic apoptotic pathway

**Figure 5:**
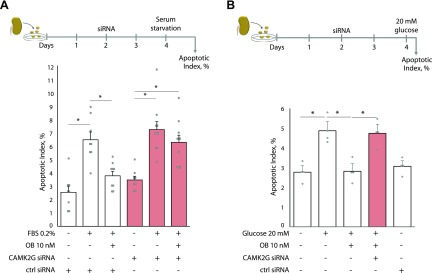
CAMK2G acts downstream of NKA, mediating protection from apoptosis. *A*, *B*) Top: Time plan of transfection of primary rat proximal tubular cells with CAMK2G or control (ctrl) siRNA followed by the indicated treatment. *A*) Top: 48 h after CAMK2G or ctrl siRNA transfection, cells were cultured with 10% (v/v) or 0.2% (v/v) FBS in the presence or absence of 10 nM ouabain for 24 h. *B*) Top: 48 h after CAMK2G or ctrl siRNA transfection, cells were exposed to normal (5.5 mM) or high (20 mM) glucose concentration in the presence or absence of 10 nM ouabain for 6 h. Nanomolar concentration of ouabain yields physiologic effects after long exposure time because ouabain binding to NKA is characterized by a slower off-rate than on-rate (68). *A*, *B*) Bottom: Apoptotic index of rat proximal tubular cells (*y* axis) as measured by TUNEL assay. At least 8 (*A*) or 4 (*B*) replicates were employed for analysis. Individual data points are represented as gray dots. OB, ouabain. *P < 0.05.

The CAMK family of proteins is highly sensitive to [Ca^2+^]_i_ oscillations. CAMK2G has been reported to specifically phosphorylate the proapoptotic Bcl-2–family protein BAD at a site that blocks its proapoptotic effect (22). Because ouabain protects primary rat proximal tubule cells from apoptosis caused by serum deprivation (10), Shiga toxin (12), excessive concentration of albumin (13), or glucose (ongoing study), we tested whether CAMK2G down-regulation abolishes the antiapoptotic effect of ouabain. Primary rat proximal tubule cells, transfected with siRNA targeting CAMK2G mRNA or with nontargeting control siRNA (Supplemental Fig. S5), were exposed to serum deprivation, a well-known trigger of apoptosis in these cells, and concomitantly treated with ouabain for 24 h (10). Ouabain protects from apoptosis serum-deprived cells treated with control siRNA but has no antiapoptotic effect on serum-deprived cells where CAMK2G was down-regulated (**Fig. 5*A***). To further test the physiologic relevance of this finding, primary proximal tubule cells were also exposed to high glucose, a well-known trigger of apoptosis and a major mediator of diabetic complications (60). Ouabain protects from apoptosis cells exposed to high glucose but not cells exposed to high glucose where CAMK2G was down-regulated (Fig. 5*B*).

## DISCUSSION

This study represents a major advancement in the understanding of the dual function of NKA as an ion pump and as a signal transducer activated by the cardiotonic steroid ouabain. Distinct domains of the NKA catalytic subunit are responsible for the ion transporting function and for the calcium-signaling process. The latter is triggered by binding of the N-terminal tail of the NKA catalytic α subunit to the N terminus of the InsP_3_ receptor (17, 18). The NKA N-terminal tail can be truncated without changing the pump-transporting function, and studies of NKA crystal structure have demonstrated that the NKA N terminus is not involved in ion transport (61). Evidence for an additional role of the NKA as a signal transducer have been accumulating since it was reported in 1996 by Peng *et al.* (62) that subsaturating concentration of ouabain induces calcium-dependent expression of early response genes in cardiomyocytes. In a series of studies, the Xie group has shown that ouabain activates SRC and MAPKs (14, 52). In the current study we did not detect SRC phosphorylation at the examined time points, which does not, however, exclude that reversible SRC phosphorylation may occur at earlier time points. Indeed, the enrichment of proteins belonging to the MAPK family in cluster 1 of early down-regulated phospho-sites and the transient up-regulation of MAPK3 (ERK1) 5 min after ouabain treatment (Fig. 3*A*) suggest that this may be the case.

Our study offers an explanation for the mechanism of control of the calcium oscillatory signal triggered by ouabain-bound NKA. Oscillations of [Ca^2+^]_i_ depend on the concerted activation of the InsP_3_ receptor and the store-operated calcium channels regulated by STIM. This interaction occurs along the junctions between the ER and plasma membrane, the same location where InsP_3_ receptors interact with NKA (63). Ouabain altered the state of phosphorylation of both STIM and the InsP_3_ receptor. STIM was phosphorylated at a site that is regulated after the release of calcium from the ER (41, 42). Dephosphorylation of the InsP_3_ receptor S1832 was predicted to enhance the opening of the InsP_3_ receptor calcium channel, thereby increasing the frequency of [Ca^2+^]_i_ oscillations.

It is well recognized that ouabain promotes cell-to-cell contacts and cell proliferation in cultured cells (8, 9, 43). There is also evidence that ouabain affects embryonic growth and development in mammals, because offspring of mice treated with anti-ouabain antibodies have lower body weight (11) and ouabain supplementation to malnourished pregnant mice prevents adverse development of the kidney in the offspring (64). Our study provides the first insight into the molecular mechanisms behind the effects of ouabain on growth and development by characterizing networks of phosphoproteins involved in regulating these processes (Supplemental Fig. S4*B*). Because organ development relies on the crosstalk between cell growth and apoptosis (65, 66), it is interesting that phosphorylation of PRKDC, a protein that regulates cellular growth and participates in DNA repair following activation of the apoptotic pathway, was one of the top up-regulated events after 10 min of ouabain treatment.

Members of the CAMK2 family of proteins are particularly sensitive to the [Ca^2+^]_i_ oscillatory signal (20, 21, 67). Here, we found that CAMK2G, which phosphorylates the proapoptotic Bcl-2 family protein BAD at a site that blocks its proapoptotic effect (22), is phosphorylated in response to ouabain and is required for ouabain ability to protect cells from apoptosis. Phosphorylation of CAMK2G upon ouabain treatment occurred on S402 and S449, 2 sites whose function is currently not known. Experiments based on site-directed mutagenesis that either mimic (S to D or E) or ablate phosphorylation (S to A or V) may shed light on their roles.

Protection from apoptosis is a well-documented downstream effect of ouabain treatment (10, 12, 13). Apoptosis is a major contributor to the progressive loss of functional renal tissue in kidney disease (60). Studies where primary rat kidney epithelial cells were exposed to serum deprivation, Shiga toxin, or a high concentration of albumin or glucose showed that ouabain protects from apoptosis by deactivating proapoptotic members of the Bcl-2 family of proteins. The capacity of ouabain to rescue primary renal epithelial cells exposed to serum deprivation or high glucose from apoptosis was lost upon CAMK2G down-regulation. This finding provides insight into the mechanism of ouabain antiapoptotic effect and may offer a much-needed novel therapeutic option to prevent the loss of functional tissue in chronic kidney disease.

In summary, this study demonstrates that ouabain activates a large-scale signaling network and that the signaling function of the NKA can be considered comparable to the signaling function of G protein-coupled receptors. Additionally, this study provides a mechanism for the origin of [Ca^2+^]_i_ oscillations triggered by ouabain-bound NKA, illustrates the coupling between slow [Ca^2+^]_i_ oscillations and CAMK2G activation, and provides an explanation for several previous observations of the effects of ouabain on cell proliferation, cell-to-cell junctions, and apoptosis. Finally, this study establishes CAMK2G as an effector of NKA-mediated protection from apoptosis.

## Supplementary Material

This article includes supplemental data. Please visit *http://www.fasebj.org* to obtain this information.

## Abbreviations

AM: acetoxymethyl
BAD: BCL2 associated agonist of cell death
[Ca^2+^]i: intracellular calcium concentration
Bcl-2: B-cell lymphoma 2
CAMK: calcium and calmodulin–dependent protein kinase
CAMK2: CAMK type II
CAMK2G: CAMK2-γ
ER: endoplasmic reticulum
FBS: fetal bovine serum
FTMS: Fourier transform-based mass spectrometer
GO: gene ontology
HCD: higher energy collision dissociation
HiRIEF: high-resolution isoelectric focusing
InsP_3_: inositol triphosphate
IPG: immobilized pH gradient
LC-MS/MS: liquid chromatography MS/MS
MS: mass spectrometry
MS/MS: tandem MS
NKA: Na^+^, K^+^–ATPase
PDB ID: Protein Data Bank identifier
phospho-motif: phosphorylation motif
phosphopeptide: phosphorylated peptide
phosphoprotein: phosphorylated protein
phospho-site: phosphorylation site
PRKDC: DNA-dependent protein kinase
siRNA: small interfering RNA
STIM: stromal interaction molecule
TMT: tandem mass tag
