# Supplementary material for: Ouabain-regulated phosphoproteome reveals molecular mechanisms for Na+, K+–ATPase control of cell adhesion, proliferation, and survival
Source: FASEB J. 2019 Jul 10;33(9):10193–206. doi: 10.1096/fj.201900445R (PMC6704450; doi:10.1096/fj.201900445R)
Supplement: Supplementary file 6 [file fj.201900445R.st1.docx]

**Supplemental Table S1.** **Number of identifications for phosphoproteomic and proteomic analyses.**

| **Phospho-proteomics analysis – Peptide and protein identifications** | | | | |
| --- | --- | --- | --- | --- |
| IPG strip pH range | No. of unique peptides† | No. of unique phospho-peptides† | No. of unique phospho-proteins† | No. of unique genes†‡ |
| 2.5-3.7 | 8,670 | 6,253 | 2,762 | 2,564 |
| 3-10 | 24,774 | 11,724 | 3,710 | 3,448 |
| **Combined analysis*** | 30,936* | 16,394* | 4,338* | 4,026* |

| **Phospho-proteomics analysis – Phospho-sites identifications** | | | | | |
| --- | --- | --- | --- | --- | --- |
| No. of identified phospho-sites | No. of phospho-sites with high confident localization (pRS score >= 95) | pSer sites | pThr sites | pTyr sites |  |
| 18,555 | 15,348 | 12,903 | 2,323 | 122 |  |

| **Proteomics analysis - Identifications** | | | |
| --- | --- | --- | --- |
| IPG strip pH range | No. of unique peptides† | No. of unique proteins† | No. of unique genes† |
| 3-10 | 53,599 | 7,740 | 7,109 |

*“Combined analysis” reports the number of unique identifications obtained employing both IPG strips, pH range 2.5-3.7 and 3-10.

†Peptides are defined as unique based on sequence and number of phosphorylations that they carry. Proteins and genes are defined as unique by Uniprot ID and gene symbol, respectively.

‡For phosphoproteomics analysis, the number of unique genes refers to genes corresponding to phosphorylated proteins only.
